# Not So Slim Anymore—Evidence for the Role of SUMO in the Regulation of Lipid Metabolism

**DOI:** 10.3390/biom10081154

**Published:** 2020-08-06

**Authors:** Amir Sapir

**Affiliations:** Department of Biology and the Environment, Faculty of Natural Sciences, University of Haifa–Oranim, Tivon 36006, Israel; amir-s@sci.haifa.ac.il; Tel.: +972-495-396-15

**Keywords:** SUMO, SUMO proteases, lipid metabolism, nuclear receptors, SREBPs, fatty acid metabolism, metabolism of cholesterol, steroid hormones

## Abstract

One of the basic building blocks of all life forms are lipids—biomolecules that dissolve in nonpolar organic solvents but not in water. Lipids have numerous structural, metabolic, and regulative functions in health and disease; thus, complex networks of enzymes coordinate the different compositions and functions of lipids with the physiology of the organism. One type of control on the activity of those enzymes is the conjugation of the Small Ubiquitin-like Modifier (SUMO) that in recent years has been identified as a critical regulator of many biological processes. In this review, I summarize the current knowledge about the role of SUMO in the regulation of lipid metabolism. In particular, I discuss (i) the role of SUMO in lipid metabolism of fungi and invertebrates; (ii) the function of SUMO as a regulator of lipid metabolism in mammals with emphasis on the two most well-characterized cases of SUMO regulation of lipid homeostasis. These include the effect of SUMO on the activity of two groups of master regulators of lipid metabolism—the Sterol Regulatory Element Binding Protein (SERBP) proteins and the family of nuclear receptors—and (iii) the role of SUMO as a regulator of lipid metabolism in arteriosclerosis, nonalcoholic fatty liver, cholestasis, and other lipid-related human diseases.

## 1. The Core Regulatory Circuit of SUMO

**Figure 1:**
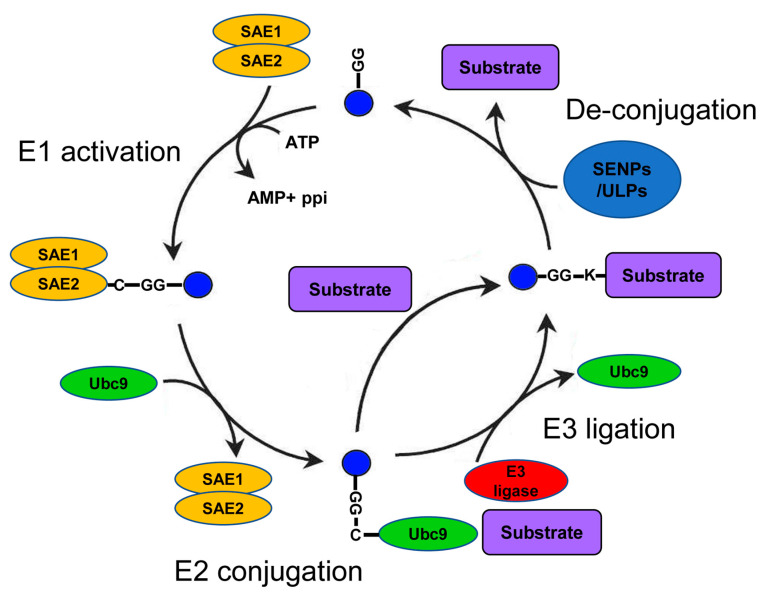
The Small Ubiquitin-like Modifier (SUMO) conjugation cycle: The names of the enzymes are based on mammalian nomenclature. After SUMO cleavage (not shown), SUMO is conjugated to a heterodimeric E1 ligase complex (SAE1/2). Next, SUMO is transferred to the E2 ligase (Ubc9). Finally, the SUMO-Ubc9 molecule forms a complex with an E3 ligase (e.g., PISA1–4) and with the target protein. This complex formation facilitates the transfer of SUMO to a specific lysine residue on the sequence of the target protein. Alternatively, SUMO conjugation to the E2 ligase, Ubc9, can be followed by the direct transfer of SUMO to the protein target independently of E3 ligase activity. Substrates can be conjugated with a single SUMO (monoSUMOylation), with multiple SUMO peptides on different lysine residues (multiSUMOylation), or with a chain of SUMO2/3 tags (polySUMOylation). SUMO-specific proteases (named SENtrin-specific Proteases (SENPs) in mammals and Ubiquitin-Like Proteases (ULPs) in nonmammalian organisms cleave SUMO from its protein substrate in order to reverse SUMO conjugation and activity.

A growing number of studies show the importance of Small Ubiquitin-like Modifier (SUMO) as a regulator of numerous cellular processes in health and disease [1,2]. Within the many types of posttranscriptional modifications of proteins (PTMs), SUMO belongs to the superfamily of ubiquitin-like modifiers that have essential functions in eukaryotes. These functions include regulation of the cell cycle [3]; transcriptional and posttranscriptional control on mRNA levels [4]; DNA damage repair [5]; regulation of signal transduction [6]; and, indirectly, protein degradation [7]. The SUMO family members are short polypeptides of about 100 amino acids that are covalently conjugated to specific lysines in the sequence of their target substrates. SUMO conjugation relies on the coordinated sequential activity of several enzymes: E1 (SUMO-activating); E2 (the SUMO-specific conjugating enzyme Ubc9); and, in some cases, E3 (SUMO ligase, e.g., the PIAS1-4 proteins) [8] (Figure 1). The final step of the cascade is the attachment of SUMO to a specific lysine residue on the substrate undergoing SUMOylation (SUMO conjugation to its protein substrate). However, in contrast to ubiquitin, SUMO proteins do not directly target proteins for degradation but rather alter substrate localization, solubility, or conformation. SUMOylation is a highly regulated and reversible process due to the activity of both positive and negative regulators of SUMO conjugation [9]. One group of proteins that plays a key role in the regulation of SUMOylation are the SUMO proteases SENPs/ULPs (SENtrin-specific Proteases/Ubiquitin-Like Proteases) [10]. Although some SENPs/ULPs play a role in the maturation of SUMO before its conjugation, the most common function of these proteases is the cleavage of SUMO from its target (de-SUMOylation) in a process counteracting SUMO conjugation.

The human genome harbors genes for five SUMO proteins (SUMO1–5), which show some nonoverlapping functions [11]. Whereas the role of SUMO4 is still not well understood, several studies have shown that the main role of SUMO1 is to regulate processes in the context of normal physiology. The activity of two additional SUMO proteins with an extremely high level of sequence similarity, SUMO2 and SUMO3 (henceforth SUMO2/3), was initially attributed to the regulation of various stress responses similar to the proposed role of SUMO5 [12,13]. However, recent findings have suggested that SUMO2/3 proteins have functions, including the regulation of specific steps of the cell cycle [14], that are unrelated to stress. Another difference between SUMO proteins in mammals is the potential to form a chain of modifiers. SUMO2/3 and 5 molecules can form chains on the targeted protein because they possess lysine residues near their amino termini, which serve as SUMO-acceptor sites. The lack of such lysines in the sequence of SUMO1 suggests that this form of SUMO is only mono-conjugated to the target. The biological significance of mono- versus chain-conjugation is still an active area of research [15]. The SUMO-conjugation pathway (E1 + E2 + E3) and SUMO-deconjugating enzymes (SENPs/ULPs) constitute the direct SUMO regulatory network, which determines the level, pattern, and dynamics of SUMOylation of target proteins [15]. Other PTM processes, like phosphorylation, acetylation, and ubiquitination, antagonize or enhance the level of SUMO conjugation. In many cases, the cross-talk between different PTM processes is the mechanism by which SUMO manifests its activity on the target protein.

## 2. Lipids—Basic Properties and Metabolism

**Figure 2:**
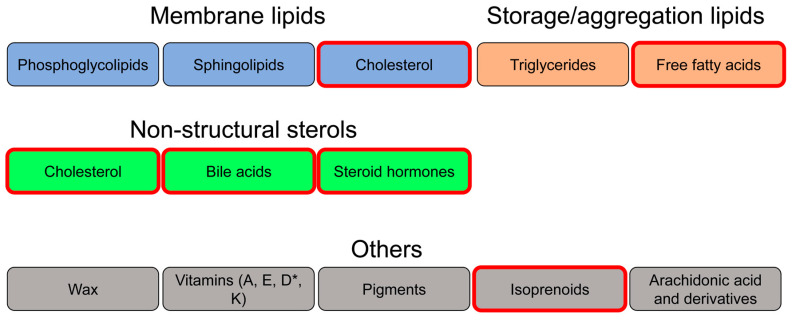
Types of lipids that are suggested to be regulated by SUMOylation: Collection and classification of lipids based on textbook literature [19]. The red frames label lipids that are suggested to be regulated by SUMOylation. These include, for example, ergosterol in yeasts [24,25,26]; isoprenoids in *C. elegans* [27]; and, in mammals, cholesterol [28], bile acids [29,30], and free fatty acids [31,32]. Lipids are grouped by their biological functions and not by their chemical properties. Cholesterol has both structural and nonstructural roles; thus, it appears in two different categories. * Vitamin D is a byproduct of cholesterol, but it is grouped with other vitamins based on its biological function.

**Figure 3:**
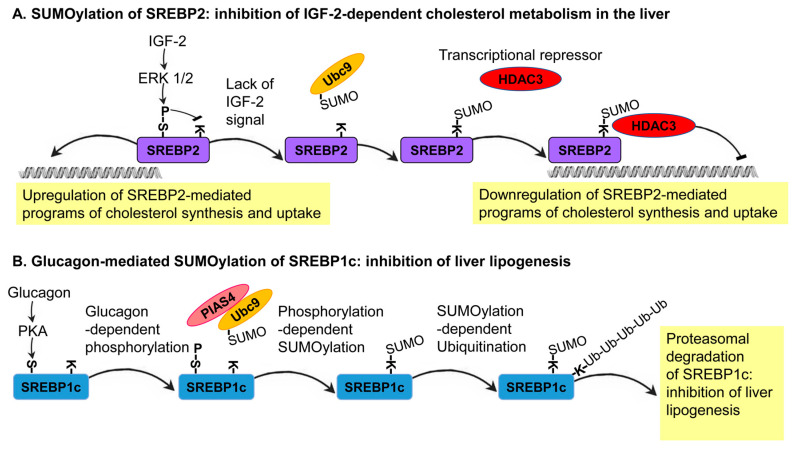
Two prominent cases of the regulation of SREBP-dependent lipid metabolism by SUMOylation: (**A**) In specific physiological conditions, the hormone Insulin-like Growth Factor 2 (IGF-2) activates two mitogen-activated protein kinases, ERK1 and ERK2, in order to phosphorylate SREBP2. This regulated phosphorylation reduces the level of SREBP2 SUMOylation, thus, leading to the upregulation of genes that encode enzymes of sterol metabolism, including the LDL receptor (LDLR), squalene synthase, and HMG-CoA synthase [28]. In the absence of the IGF-2 signal, SREBP2 SUMOylation leads to the inhibition of the transcriptional activity of SREBPs through the recruitment of a corepressor complex that includes the histone deacetylase 3 (HDAC3) [28]. (**B**) In fasting conditions, the secretion of the mouse fasting hormone, glucagon, from the pancreas activates Protein Kinase A (PKA), which phosphorylates SREBP1c on serine 308. This phosphorylation increases the levels of SREBP1c SUMOylation, leading to its ubiquitination-dependent degradation. SREBP1c degradation results in the inhibition of lipogenesis in order to switch the metabolic state of the organism toward catabolism [33]. This mechanism was further elucidated by the overexpression of the SUMO E3 ligase, PIAS4, in obese db/db mice, which leads to the inhibition of lipid synthesis. Moreover, the suppression of PIAS4 activity in lean mice triggers the expression of SREBP1c target genes that stimulate hepatic lipogenesis [33].

The defining characteristic of all lipids is their solubility in nonpolar organic solvents but not in water [16]. Based on the degree of solubility in organic solvents, lipids are subdivided into groups ranging from highly hydrophobic to amphiphilic molecules. Chemically and structurally, lipids can be subdivided into eight classes: fatty acids (FA), glycolipids, glycerophospholipids, sterols and sterol derivatives, sphingolipids, prenol lipids, glycolipids, and polyketides [17,18]. Another type of classification of lipids relies on the function of lipids within the complex metabolism of the cell [19]. Lipids have a key structural role in constituting the membranes that surround and, thus, define the cell and its organelles [20]. In addition, specific types of lipids have various nonstructural functions, for example, fatty acids used for energy storage [21] and steroid hormones for signaling [22]. The functional classification of lipids includes the structural phosphoglycolipids, sphingolipids, and cholesterol in cellular membranes; triglycerides and free fatty acids as storage lipids; bile acids, steroid hormones, and other nonstructural sterols; and wax, vitamins, pigments, isoprenoids, and arachnoid acid (Figure 2). A complex network of enzymes facilitates the synthesis, modification, and breakdown of the different types of lipids within the cell [23]. The activity of such enzymes of lipid metabolism can be regulated by many different transcriptional and posttranslational mechanisms. One posttranslational mechanism that has emerged in recent years as an important regulatory mechanism of lipid metabolism is the conjugation of SUMO to target proteins. For example, the SUMOylation of key master regulators of lipid metabolism, the Sterol Regulatory Element Binding Proteins (SREBPs), coordinate lipid homeostasis with the physiological state of the organism (Figure 3) [1,2,3,4,5,6,7,8,9,10,11,12,13,14,15].

## 3. SUMO and Lipid Metabolism in Fungi and Invertebrates

Similar to ubiquitin, SUMO is a hallmark of eukaryotes; SUMO modification has been identified and studied in major groups of eukaryotes, including fungi, plants, invertebrates, and vertebrates. Most of what we know about the function of SUMO is based on studies of model organisms in these taxa. Importantly, the general principles of SUMO activity and its regulation are conserved from yeasts to humans [34]. However, the SUMO regulatory network has fewer components in yeasts and invertebrates than in mammals. There are five SUMO proteins in humans but the yeast model organism, *Saccharomyces cerevisiae* (*S. cerevisiae*); the nematode model, *Caenorhabditis elegans* (*C. elegans);* and the insect model, *Drosophila melanogaster* (*D. melanogaster*), have only one SUMO protein. In addition to a smaller regulatory network of SUMOylation, these experimental systems are amenable for large-scale genetic manipulation; thus, the study of these model organisms holds the promise to uncover novel and conserved roles of SUMO as a regulator of lipid metabolism.

## 4. Yeast—The Regulation of Lipid Metabolism by SUMO in Eukaryotic Protozoa

In the primary model organism of fungi, the budding yeast *S. cerevisiae* proteomic screens conducted to find SUMOylated proteins in vivo by immunoprecipitation (IP) and mass-spectrometry analysis (MS) have identified many proteins that are involved in lipid metabolism. For example, among the candidate targets for SUMOylation are many of the ERG proteins of the mevalonate pathway that are responsible for the synthesis of the primary sterol of fungi, ergosterol. The list of ERG proteins that are candidates for SUMOylation includes ERG3, ERG7, ERG9, and ERG27 [24]; ERG10 and ERG13 [25]; and ERG20 [26]. The mevalonate pathway is an evolutionarily conserved pathway among eukaryotes that facilities the synthesis of many important lipids from acetyl-CoA. The main branch of the mevalonate pathway divides into several subbranches that facilitate the synthesis of specific metabolites. In addition to sterols, these lipids include, for example, prenyl moieties required for the prenylation of proteins and ubiquinone—an electron carrier that functions in the mitochondrial electron transfer chain [35]. Some of the enzymes identified in the proteomic screens as candidate targets for SUMOylation are enzymes of the main branch of the mevalonate pathway including the first dedicated enzyme of the pathway, ERG13. Other putative targets are enzymes of the ergosterol synthesis subbranch, such as ERG27. Thus, SUMOylation is predicted to regulate both ergosterol synthesis and the metabolism of other subbranches of the mevalonate pathway—for example, the synthesis of electron carriers that affect mitochondrial function. In addition to the direct conjugation of SUMO to specific ERG proteins, SUMOylation is required for the nuclear localization of Multicopy suppressor of GAm1 (MGA2), a master regulator of fatty acid and sterol metabolism in *S. cerevisiae* [24]. In a *ubc9* mutant background, the nuclear localization of the MGA2 transcription factor is impaired. This finding suggests that transcriptional regulation is another mechanism by which SUMO may control lipid metabolism in *S. cerevisiae*. The physiological significance of the regulation of lipid metabolism by SUMO is not entirely clear but the synthetic lethal phenotypes of *erg8* or *erg11* mutations in the background of deletions in two E3 ligases, *siz1/2*, suggest a functional link between SUMO and ergosterol synthesis [24,36,37]. The regulation of the synthesis of sterols and other products of the mevalonate pathway by SUMO in *S. cerevisiae* represents a mode of regulation conserved among other eukaryotes including *C. elegans* and mammals as will be discussed below.

## 5. Nematodes and Flies—The Role of SUMO in Regulated Lipid Metabolism of Invertebrates

The role of SUMO in lipid metabolism of invertebrates was studied primarily in the two model organisms of invertebrates, *C. elegans* and *D. melanogaster* [38]. Notably, the mevalonate pathway of these two organisms has lost the ability to synthesize cholesterol [39,40] but still synthesizes several essential lipids [24]. Thus, although the proposed role of SUMO in the regulation of sterol synthesis in *S. cerevisiae* [24,25,26], [24] is not conserved in these invertebrates SUMO can still regulate other branches of the mevalonate pathway.

In *C. elegans*, several large-scale proteomic screens of IP and mass-spectrometry analysis have resulted in lists of candidate targets for direct SUMO conjugation [41,42]. These candidates include, for example, the FAT-1 (omega-3 fatty acyl desaturase) and FAT-2 (Oleate/linoleate desaturase) proteins [41] as well as the FASN-1 (Fatty Acid SyNthase) enzyme [42]. However, the biological significance of SUMO conjugation to enzymes of lipid metabolism identified in these screens is still largely unknown. A more focused study has shown that the enzyme HMG-CoA Synthase (HMGS-1) of *C. elegans* is SUMOylated in an age-dependent manner [27]. HMGS-1 is the first dedicated enzyme of the mevalonate pathway that, in *C. elegans*, facilitates the synthesis of essential lipids, such as ubiquinone—an electron carrier in the mitochondrial electron transfer chain [35,43,44]. The activity of HMGS-1 is suggested to be balanced by inhibitory SUMOylation and activating de-SUMOylation of HMGS-1 by the SUMO protease ULP-4. In accordance with this model, the HMGS-1 protein is over-SUMOylated in *C. elegans* with an *ulp-4* loss of function mutation [27]. Importantly, some of the deleterious phenotypes of *ulp-4* mutant worms, such as impaired development and growth, can be suppressed by the supplementation of mevalonate that upregulates mevalonate pathway metabolism. This finding suggests that SUMOylation of ULP-4 is a mechanism used to downregulate the metabolism of the mevalonate pathway with age. Notably, HMGS-1 SUMOylation is predicted to be evolutionarily conserved from yeasts to humans [27], highlighting a possible general mechanism in eukaryotes of age-dependent regulation of lipid metabolism by SUMOylation.

In *D. melanogaster*, SUMO, encoded by the *smt3* gene, is required for fatty acid metabolism and the synthesis of the steroid hormone, ecdysone, which controls larval development and metamorphosis [45]. In the cells of the prothoracic gland (PG), which is responsible for ecdysone synthesis, the knockdown of SUMO decreases the level of ecdysteroid synthesis, leading to a block of larval–pupal transition [45]. Fewer sterol-containing lipid droplets in prothoracic gland cells suggest that SUMO plays a role in the uptake of cholesterol, the precursor of the hormone ecdysone, into these cells. Thus, the effect of SUMO on ecdysone synthesis is suggested to stem, at least in part, from the regulation of cholesterol uptake. Molecularly, SUMO conjugation activates a transcription factor from the nuclear receptor family, Ftz-f1, and subsequently a downstream transcription factor, Snmp1, that is suggested to be responsible for the uptake of cholesterol from lipid droplets [46]. Notably, the overexpression of Snmp1 can rescue the phenotype of lipid droplets loss of *smt3* and *Ftz-f1* knockdowns, demonstrating that Snmp1 is the primarily transcriptional target of SUMOylated Ftz-f1. The function of Ftz-f1 was also linked to the metabolism of fatty acids. The exact mechanism underlying this activity as well as the target genes of Snmp1 that regulate ecdysone levels are yet-to-be characterized. Control of fatty acid and sterol metabolism by the SUMOylation of nuclear receptors in *D. melanogaster* is highly conserved in mammals as will be discussed below.

## 6. SUMO and Lipid Metabolism in Mammals

### SUMO Proteins Control Lipid Metabolism through Transcriptional Regulation

Of many types of lipids in mammals (Figure 2), cholesterol and fatty acids are the most studied. One characteristic of cholesterol is that it can be either produced from simple cellular building blocks by the mevalonate pathway or acquired through diet [47,48,49]. Similarly, most fatty acids have two sources—*de novo* synthesis or intestinal uptake [50], although a few “essential” fatty acids cannot be synthesized by most animals [51]. Decades of research have discovered numerous structural, metabolic, and regulative roles of cholesterol and fatty acids in mammals. Two prominent examples are the control of membrane fluidity and membrane rafts dynamics through the regulated composition of cholesterol and fatty acids [52,53]. Changes in the levels of cholesterol and fatty acids, deficiencies or excesses, often have fundamentally negative consequences at the cellular and organismal levels. Thus, a fine balance between the biosynthesis of lipids, the level of their absorption in the intestine, and the degree of their transport to and from storage organs must be maintained [54]. Perturbations in this homeostatic control can lead to widespread pathologies; for example, a high level of Low-Density Lipoprotein (LDL) particles in the blood is considered as the primary cause of cardiovascular disease, one of the primary medical challenges of our time [55].

## 7. SREBP Proteins

A group of primary master regulators of fatty acid and cholesterol metabolism consists of the transcription factors Sterol Regulatory Element Binding Proteins (SREBPs) [54,55,56]. Importantly, the SUMOylation of SREBPs described below is the most direct link between SUMO and lipid metabolism. The SREBP proteins constitute a distinct subclass in the basic helix-loop-helix leucine zipper (bHLH-Zip) family of transcription factors [57]. In mammals, two genes (SREBP1 and SREBP2) encode three functionally distinct isoforms (SREBP1a, SREBP1c, and SREBP2) [58]. SREBP1a and SREBP1c coordinate the metabolism of fatty acids, triglyceride, and phospholipids. SREBP1a is expressed in proliferating tissues, whereas SREBP1c is expressed in liver and adipose tissues [58,59]. In contrast, the SREBP2 transcription factor is primarily dedicated to the regulation of cholesterol synthesis; thus, it is more ubiquitously expressed [60]. Reduction in the level of cholesterol activates a compensatory mechanism of SREBP cleavage and subsequently nuclear localization of the transcriptional activating domain of the proteins. The nuclear localization of the cleaved portion of SREBPs leads to the synthesis of genes encoding different enzymes of cholesterol and fatty acid metabolism [54,55,56,58]. In addition to this mechanism, the activity of the cleaved SREBPs is regulated by several types of PTM, including ubiquitination, phosphorylation, and SUMOylation [28,61].

SREBP1a was shown to be SUMOylated on lysine 123 and 418, whereas lysine 464 is SUMOylated on SREBP2. The Ubc9-mediated conjugation of SUMO1 to these sites results in the inhibition of the transcriptional capacity of these SREBP proteins; thus, highlighting the inhibitory role that SUMOylation plays on SREBP protein activity [61]. A follow-up study in different cell lines, including the human liver-derived cell line, HepG2, found that SREBP2 SUMOylation leads to the inhibition of the transcriptional activity of SREBPs through the recruitment of a corepressor complex that includes the histone deacetylase 3 (HDAC3) [28]. As in many other reported cases of SUMOylation, at a given time, only a small fraction of the SREBP2 that forms a complex with HDAC3 is SUMOylated. Thus, SUMOylation does not inhibit the transcriptional activity of SREBPs directly but functions as a molecular link between SREBPs and HDAC3. In specific physiological conditions, the hormone IGF-2 activates two mitogen-activated protein kinases (MAPKs), ERK1 and ERK2, in order to phosphorylate SREBP-2 at serine 455. This regulated phosphorylation reduces the level of SREBP2 SUMOylation, thus leading to the upregulation of genes that encode enzymes of sterol metabolism, including the LDL receptor (LDLR), squalene synthase, and HMG-CoA synthase [28]. In this system, a growth-hormone-mediated and ERK-dependent phosphorylation of SREBPs antagonizes its SUMOylation as part of a molecular switch mechanism coordinating cholesterol metabolism with the physiological state of the organism (Figure 3A).

An inhibitory effect of SUMOylation was also described for SREBP1c. A study of primary hepatocytes in culture and mouse models has shown that the SUMOylation of SREBP1c at lysine 98 by the SUMO E3 ligase, PIAS4, leads to the suppression of the hepatic lipogenic programs with fasting [33]. During fasting, the secretion of the fasting hormone, glucagon, from the pancreas activates a protein kinase, PKA, which phosphorylates SREBP1c on serine 308. This phosphorylation increases the levels of SREBP1c SUMOylation and consequently its ubiquitination-dependent degradation. SREBP1c degradation results in the inhibition of lipogenesis, switching the metabolic state of the organism toward catabolism [33]. This mechanism was further exemplified by the overexpression of the SUMO E3 ligase, PIAS4, in obese db/db mice, which leads to the inhibition of lipid synthesis. In support of this finding, the suppression of PIAS4 activity in lean mice triggers the expression of SREBP1c target genes that stimulate hepatic lipogenesis [33]. This in vivo system further demonstrates the role of SUMO as an SREBP1c-dependent inhibitor of lipogenesis (Figure 3B). Notably, the two outcomes of SREBPs SUMOylation—transcriptional repression (SREBP2) and induced degradation (SREBP1c)—lead to the same result: inhibition of SREBP-dependent transcription.

## 8. Nuclear Receptors

SUMOylation plays a cardinal role in the regulation of the activity of many nuclear receptors (NRs)—a superfamily of sensors that respond to changes in the concentration of numerous small molecules. The canonical mechanism of NR activity involves the binding of a ligand, for example, a steroid hormone, to its nuclear receptor in the cytoplasm or the nucleus of the cell. This binding induces a conformational change that consequently alters the subcellular location and/or the transcriptional activity of the nuclear receptor [62,63]. Seventeen nuclear receptors were reported to be SUMOylated [64], but this number is expected to increase considerably with the development of new approaches to identify SUMOylated proteins.

**Figure 4:**
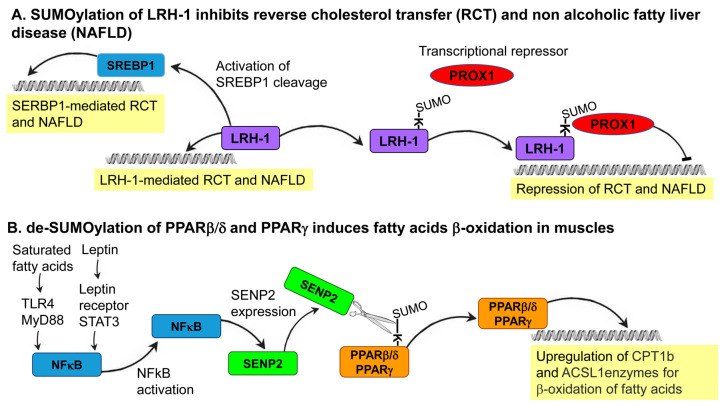
Two cases-in-point of the regulation of lipid homeostasis by the SUMOylation of nuclear receptors (NRs). (**A**) In a mouse model system, Liver Receptor Homolog-1 (LRH-1) coordinates programs of reverse cholesterol transport (RCT) and cholesterol metabolism. SUMOyaltion of LRH-1 results in the recruitment of the transcriptional-repressor PROspero-related homeoboX 1 (PROX1) to the LRH-SUMO protein. The LRH/PROX1 complex inhibits the expression of genes that function in RCT and in cholesterol and bile acid excretion [29]. UnSUMOylated LRH-1 also promotes SREBP1 activation and the execution of lipogenesis programs in the liver, which leads to the development of nonalcoholic fatty liver disease [66]. (**B**) In the mouse skeletal muscle-derived cell line, C2C12, the supplementation of saturated fatty acids activates the toll-like receptor 4 (TLR4) and its adaptor MyD88 [31]. This activation results in NFkB-mediated upregulation of the SUMO protease SENP2, which sheds SUMO from PPARβ/δ and PPARγ. This deconjugation leads to the transcriptional upregulation of fatty-acid oxidation-associated enzymes, such as carnitine palmitoyl transferase-1 (CPT1b) and long-chain acyl-CoA synthetase 1 (ACSL1). SENP2 upregulation was also demonstrated following the treatment of C2C12 cells with the hormone leptin that activates the STAT3 transcription factor [71]. In SENP2 knockout mice, leptin treatment does not upregulate CPT1b and ACSL1 levels as well as fatty acid β-oxidation, demonstrating the in vivo significance of the link between the SUMOylation of PPAR proteins and lipid metabolism [71].

One of the best examples of a SUMO-regulated nuclear receptor that controls lipid metabolism is the Liver Receptor Homolog-1 (LRH-1)—a central regulator of cholesterol homeostasis. The primary role of LRH-1 is to facilitate the process of reverse cholesterol transport (RCT) from peripheral tissues to the liver and the subsequent excretion of cholesterol and bile acids from the liver to the alimentary system. In a mouse model system, blocking the SUMOylation of lysine 289 in LRH-1 was shown to result in the upregulation of a set of genes related to cholesterol metabolism and an increased level of cholesterol and bile acid excretion [29]. Mechanistically, SUMO conjugation results in the binding of the transcriptional-repressor PROspero-related homeoboX 1 (PROX1) to LRH-1, and the LRH/PROX1 complex inhibits the expression of genes that function in bile acids and cholesterol metabolism (Figure 4A). Another possible mechanism by which SUMO may inhibit LRH-1 function is the accumulation of SUMOylated LRH-1 in promyelocytic leukemia protein nuclear bodies, which prevent the access of LRH-1 to the chromatin [65]. These two proposed mechanisms underlie SUMO as a negative regulator of LRH-1 function and, consequently, the RCT process of cholesterol and bile-acids excretion [29]. In addition, introducing to the mouse genome a genetically engineered variant of LRH-1 that cannot be SUMOylated results in a high level of SREBP1 processing. An increase in the level of SREBP1 cleavage facilitates the execution of lipogenesis programs in the liver, which leads to the development of nonalcoholic fatty liver disease [66]. This observation demonstrates how the direct effect of SUMO conjugation on one protein, LRH-1, results in the modified activity of another master regulator of lipid metabolism, SREBP1.

Additional regulators of RCT that might be subjected to functional control by SUMO are the Liver X Receptor proteins (LXRs). The two isoforms of this protein, LXRα and LXRβ, are central regulators of different aspects of sterol metabolism (review in [67]). These proteins can bind directly to the DNA and can function as transcription factors but also can act as co-activators/repressors of other transcription factors. In cases of impaired cholesterol homeostasis, the LXR proteins transcriptionally upregulate genes that protect cells from cholesterol overload. For example, a high level of cholesterol in peripheral cells (e.g., macrophages) results in the activation of LXRs, which facilities the RCT process [68]. In addition to the regulation of cholesterol homeostasis, LXRs are regulators of inflammation. In human HeLa cells and mouse-derived primary macrophages, the conjugation of SUMO2/3 to LXRβ was demonstrated. This conjugation was reported to switch LX Rβ activity from executing pro- to anti-inflammatory programs [69]. In contrast to inflammation, it is yet-to-be-determined whether SUMO plays a direct role in the regulation of cholesterol metabolism by LXRs or whether this is a SUMO-independent but PIAS1-dependent process as suggested by Zhang et al. [70].

The nuclear receptor Steroidogenic Factor 1 (SF-1) is another nuclear receptor that plays a role in the regulation of lipid metabolism. Several studies have shown that Ubc9-dependent SUMO1 conjugation at lysine 119 and 194 can repress the steroidogenesis activity of SF-1 in cells of the adrenal glands and gonads [72,73,74,75]. However, another study suggests that SUMOylation can promote the transcriptional-activation function of SF-1 in normal conditions but that this modification can lead to ubiquitin-mediated degradation of SF-1 once the cells are starved [76]. The significance of SF-1 SUMOylation in terms of the expression of SF-1 target genes in the context of lipid metabolism is yet-to-be-addressed.

Another nuclear receptor that is regulated by SUMO is the Pregnane X Receptor (PXR), which coordinates programs of fatty acid, cholesterol, and lipoprotein metabolism in the liver and the intestine [77]. In HeLa cells and mouse primary hepatocytes, SUMO1 and SUMO2/3 were shown to be conjugated to the PXR protein [78,79]. These conjugations are antagonized by the activity of several SENP proteases [80]. Mechanistically, SUMO1 conjugation was suggested to enhance PXR transcriptional capacity [78] and to inhibit its degradation by the ubiquitin-proteasome system, thereby resulting in PXR activation [80]. In contrast, the conjugation of SUMO2/3 results in the ubiquitination and degradation of PXR, demonstrating that different SUMO molecules can have opposite effects on the same target. Recent studies in both primary cultures of mouse hepatocytes and cell-based assays but suggest that the SUMOylation of PXR might inhibit its transcriptional activity [81]; thus, highlighting, as in the case of SF-1, the context-dependent outcome of SUMOylation of NRs. The effect of SUMOylation on the transcriptional activity of PXR was determined for inflammation-related genes. It will be interesting to test the effect of PXR SUMOylation on genes of lipid metabolism that are also regulated by PXR [77].

The nuclear receptor Farnesoid X Receptor (FXR) is a master regulator of inflammation and bile-acid homeostasis in the liver, but in recent years, its function has also been attributed to important aspects of the metabolism of the intestine [82]. A cumulative body of evidence supports the notion that SUMOylation negatively regulates FXR activity. In the HepG2 liver-derived cell line, SUMO is conjugated to lysine 122 and 275 of the activation and ligand-binding domains of FXR [83]. The inhibition of FXR SUMOylation augmented the activation of the promoters of genes related to bile metabolism, suggesting that SUMO conjugation inhibits FXR-dependent transcription. Moreover, in liver cells derived from an obese mouse model, increased acetylation of lysine 217 inhibits the SUMOylation of FXR. Increased acetylation of FXR leads to the upregulation of inflammatory transcriptional programs [84], demonstrating the inhibitory effect of SUMOylation on FXR activity. Linking these observations with diseases of lipid metabolism is a recent study showing that the SUMOylation of FXR is gradually enhanced in the process of liver fibrosis [85]. Mechanistically, SUMOylation inhibits FXR activation and, consequently, the expression of the FXR target gene *perilipin-1,* which plays a role in the prevention of liver fibrosis. The forced activation of FXR, concomitant with the inhibition of SUMOylation, suppressed liver fibrosis, highlighting the therapeutic potential of inhibition of F XR SUMOylation in specific contexts [85].

Three well-characterized nuclear receptors that coordinate lipid metabolism are the Peroxisome Proliferator-Activated Receptors, alpha to gamma (PPARα-γ) [86]. The alpha and beta/delta genes give rise to one protein isoform per gene, whereas the gamma gene encodes three different isoforms. In the human and monkey-derived cell lines, HuH-7 and COS-7 respectively, the conjugation of SUMO1 to PPARα on lysine 185 by the activity of Ubc-9 and PIAS4 results in the recruitment of a corepressor and the differential expression of PPARα target genes [87]. Notably, this SUMOylation is antagonized by ligand binding, suggesting that SUMOylation is a switch-off mechanism ensuring ligand-specific gene expression. Another mechanism was proposed based on the study of PPARα in mouse liver, in which ligand binding induces a conformational change that exposes a SUMOylation site at lysine 385 of PPARα to UBC9 and PIAS1 of the SUMO-conjugating system [88]. The SUMOylation of lysine 385 promotes the recruitment of several proteins including a DNA methyltransferase, DNMT3, that facilitates through histone methylation transcriptional repression of PPARα target genes. One of the repressed target genes is *cyp7b1* for which the protein product inhibits synthesis of testosterone from its precursor, dehydroepiandrosterone (DHEA) [88]. This study further shows that, in the liver of female mice, sex-specific SUMOylation of PPARα and subsequentially downregulation of the CYP7B1 enzyme promote the synthesis of testosterone. Lack of PPARa SUMOylation in the male results in the upregulation of CYP7B1 that inhibits the synthesis of testosterone from DHEA in the liver. Inhibition of testosterone synthesis in the male liver results in a high level of DHEA in the circulation that is available for testosterone synthesis by the testis. Notably, this mechanism highlights the role of SUMO as a regulator of sex-specific lipid metabolism.

Accumulating data suggest that the activity of the PPARβ/δ protein is also regulated by SUMOylation. In the mouse skeletal muscle-derived cell line, C2C12, the supplementation of saturated fatty acids, such as palmitate, activates the toll-like receptor 4 (TLR4) and its adaptor MyD88 [31]. Activation of the TLR4–MyD88 axis, highly specific to saturated fatty acids, activates the transcription factor NFkB. Activation of NFkB leads to the expression of SENP2, which sheds SUMO from PPARβ/δ and PPARγ proteins. This SENP2-dependent deconjugation of SUMO activates the PPARβ/δ and PPARγ proteins, leading to the transcriptional upregulation of fatty-acid oxidation-associated enzymes, such as carnitine palmitoyl transferase-1 (CPT1b) and long-chain acyl-CoA synthetase 1 (ACSL1). The upregulation of CPT1b and ACSL1 results in fatty acid β-oxidation in the mitochondria (Figure 4B). Supporting this proposed mechanism in cultured cells is the muscle-specific overexpression of SENP2 that results in the upregulation of *cpt1b* and *acsl1* transcription in muscles of mice fed with a high-fat diet (HFD). Importantly, the overexpression of SENP2 in muscles alleviates HFD-induced obesity and insulin resistance, demonstrating the biological significance and therapeutic potential of SENP2 activity in vivo [31]. SENP2 upregulation was also demonstrated following the treatment of C2C12 cells with the hormone leptin that activates a Signal Transducer and Activator of Transcription 3 (STAT3) transcription factor [71]. STAT3-dependent upregulation of SENP2 keeps PPARβ/δ and PPARγ proteins at the unSUMOylated active state leading to the upregulation of CPT1b and ACSL1 and subsequentially fatty acid β-oxidation. Consistent with this proposed mechanism is the finding that leptin treatment does not upregulate CPT1b and ACSL1 as well as fatty acid β-oxidation in SENP2 knockout mice [71].

The activity of the three isoforms of the PPARγ protein (γ1-3) was also shown to be regulated by SUMOylation. As in the case of the other PPARs, PPARγ proteins play a dual role in the regulation of lipid metabolism and inflammation that probably represents a genuine link between these two processes. However, because the activity of PPARγ proteins is very context-dependent, the regulation of inflammation and lipid metabolism may not completely overlap. From the growing body of data about the role of SUMO in the regulation of the activity of PPARγ proteins, several reports link PPARγ SUMOylation with the regulation of the differentiation of adipocytes and with lipid synthesis in mature adipocytes. In NIH3T3 cells, SUMOylation represses the transactivation of PPARγ2, which mediates the differentiation of these cells to adipocytes [89]. In muscle-derived C2C12 cells, overexpression of the SUMO protease SENP2 results in the expression of genes of lipid metabolism in a PPARγ2-dependent manner [90]. This finding demonstrates that, in some cell lineages, SUMOylation is required to suppress PPARγ2-mediated programs of adipocyte differentiation. However, a recent study in mice showed that SUMO1-knockout animals had smaller and fewer adipocytes [91]. The conjugation of SUMO1 was shown to enhance the binding of an agonist to PPARγ2 and the transcription of genes involved in adipocytes differentiation and lipid synthesis, providing a plausible mechanism of SUMO1-dependent activation [90].

In addition to the regulation of the activity of NRs by direct conjugation, a growing body of data suggests that SUMOylation can affect the activity of NRs indirectly through modification of proteins of their regulatory network. One type of indirect regulation is the SUMOylation of NRs that form heterodimers with other NRs that are regulators of lipid metabolism. For example, critical aspects of PPAR activity are manifested by their interaction with two NRs, the Retinoid X Receptor (RXR) and the Peroxisome proliferator-activated receptor Gamma Coactivator 1 (PGC-1). The SUMOylation of the RXRα on lysine 108, located in its transcriptional activation domain, was suggested to attenuate the transcriptional activity of the RXRα homodimer and the activity of the RXRα-PPARγ heterodimeric complex [92]. The removal of SUMO1 from RXRα by SENP6 activity results in the upregulation of RXRα-PPARγ activity. In COS-7 and HeLa cells, another nuclear receptor partner of the PPAR proteins, PGC-1α, undergoes SUMO1 conjugation on lysine 183 of the transcriptional activation domain [93]. This conjugation, dependent on PIAS1 and PIAS3 and reversed by the activity of SENP1 and SENP2, inhibits the function of PGC-1α as a coactivator of PPARγ-dependent transcription. Thus, an exciting topic for future investigation is the effect of RXRα and PGC-1α SUMOylation on the function of PPARγ proteins as regulators of lipid metabolism.

The indirect effect of SUMO on the activity of PPAR proteins can also be executed by partners that are not NRs. For example, in the mouse-derived cell line, 3T3-L1, SENP2 activation promotes differentiation of the cultured cells to adipocytes [94]. SENP2 deconjugates SUMO off the CCAAT-Enhancer-Binding Protein beta (C/EBPβ). This deconjugation leads to the stabilization of C/EBPβ and consequentially to the expression of two targets of C/EBPβ, the transcription factors PPARγ and C/EBPα that drive adipocyte differentiation [94]. Another mechanism that ensures that programs of lipid metabolism will not take place in non-adipocyte cells [95] is the SUMOylation of the transcription factor Krüppel-Like Factor 5 (KLF-5). In COS-7 cells, the Ubc9-dependent conjugation of SUMO1 to lysines 162 and 209 in the sequence of KLF-5 results in the transrepression of PPARβ/δ activity and the downregulation of genes related to lipid oxidation [96]. Notably, the SUMOylation of another KLF protein, KLF-4, was reported to promote adipocyte differentiation [95], highlighting a possible general role of SUMOylation and deSUMOylation of KLF transcription factors in the regulation of adipocyte differentiation.

Similar indirect regulation of PPARγ by SUMO was also suggested to take place in fully differentiated adipocytes. Studies of differentiated adipocytes from a SENP2-knockout mouse model revealed increased SUMOylation of the SET Domain Bifurcated 1 protein, SETDB1, that binds to the promoters of PPARγ and C/EBPα genes and suppresses their expression through the modification of histone methylation [97]. The downregulation of PPARγ and C/EBPα expression results in a subsequent decrease in the transcription of target genes related to lipid metabolism in fully differentiated adipocytes [96].

SUMOylation is only one of many posttranslational modifications of NRs. These modifications often antagonistically or synergistically affect the level of NR activation. Thus, a complete understanding of the effects of SUMOylation on the activity of NRs has to take into account its interactions with other PTMs. A case in point is the phosphorylation of serine 112 in PPARγ2 that was shown to be antagonistic to the SUMOylation of lysine 107. This SUMOylation inhibits the transcriptional activity of PPARγ2; thus, PPARγ2 phosphorylation is suggested to function as a molecular switch that positively regulates PPARγ2 function in lipid metabolism [98]. This mechanism probably represents a general principle of regulated SUMOylation by different signaling pathways in order to coordinate the physiological state of the organism with the activity of NRs.

## 9. Regulation of Lipid Metabolism by the SUMOylation of Lipid-Modifying Enzymes

Compared to the well-characterized role of SUMO in the regulation of transcriptional programs, it remains largely unknown whether SUMO regulates lipid homeostasis through the direct conjugation of enzymes of lipid metabolism. One primary challenge in the study of direct modification of enzymes of lipid metabolism is the need to develop a specific metabolic assay for every enzyme of interest. Nevertheless, several large-scale screens for proteins that are directly conjugated by SUMO in mammals have identified enzymes of lipid metabolism as candidate targets for SUMO conjugation [99,100,101,102,103,104]. For example, different members of the perilipin group of proteins that play a role in adipocyte differentiation and lipid-droplets metabolism [105] were identified as putative direct targets for SUMOylation [104]. The functional significance of the direct SUMOylation of enzymes of lipid metabolism in mammals is an exciting topic for future investigation.

## 10. SUMO and Metabolic Diseases of Lipid Homeostasis

### 10.1. SUMO, NRs, and Arteriosclerosis

NRs control many disease-related processes, including the regulation of inflammation and lipid metabolism. A growing body of research links SUMO with arteriosclerosis through its proposed effect on the regulation of inflammation. The most well-characterized effect of SUMO is the activity of this protein on one of the master regulators of inflammation, NFκB, and on many NRs, including LRH-1, PPARs [106], PXR [80], and FXR [107]. However, as discussed above, these NRs are also regulators of lipid metabolism; thus, the link between SUMO and arteriosclerosis may stem from regulated lipid metabolism. For example, in an LDLR-deficient mouse model of arteriosclerosis, disturbed blood flow causes SENP2 downregulation and an increase in the level of SUMOylation of the MAPK protein ERK-5 [108]. The SUMOylation of ERK5 leads to the downregulation of PPAR activity and, consequentially, to atherosclerotic lipid accumulation and plaque formation [108]. Whether this accumulation of lipids stems exclusively from the dysregulation of inflammatory programs or also from changes in enzymes of lipid metabolism is an important topic for future investigation.

### 10.2. SUMO and Nonalcoholic Fatty Liver Disease

A recent study has highlighted a possible connection between SUMO and Nonalcoholic Fatty Liver Disease (NAFLD), which is characterized by an excessive accumulation of lipids in hepatocytes [109]. The SUMO protease SENP3 was upregulated in the liver of NAFLD patients and rats fed on a high-fat diet. Importantly, following free fatty acid treatment, lipid accumulation in human hepatocytes was markedly reduced by a SENP3-siRNA and increased by SENP3 overexpression [109]. This functional link between de-SUMOylation and the accumulation of lipids was further supported by the finding that SENP3 positively regulates the expression of several proteins that are involved in the transport of lipids to the cell, such as Apolipoprotein E [109]. Although the exact mechanistic basis of the function of SENP3 in NAFLD is yet to be characterized, this finding draws a causal link between SUMOylation, dysregulated lipid metabolism, and NAFLD.

## 11. Cancer

Several recent studies have suggested a possible role for SUMO as a regulator of lipid metabolism in specific types of cancers. One such study showed that the tumor-suppressive function of PPARγ is associated with its regulation of lipid metabolism [110]. The activation of PPARγ dramatically induced de novo lipid synthesis as well as β-oxidation of fatty acids in lung cancer cells, both in vitro and in vivo [111]. Among the genes that were shown to be differentially expressed in a PPARγ-dependent manner in these cancer cells are genes involved in lipid synthesis, lipid uptake, triglyceride metabolism, and β-oxidation. The SUMOylation of lysines 107 and 395 on PPARγ suppresses its lipid-dependent tumor-suppressive activity [111]. Another study linking SUMO activity with dysregulated-lipid metabolism in cancer shows that, in specific human-derived lung-cancer cells, Ubc9 upregulation mediates the conjugation of SUMO2 to the protein Fatty-Acid SyNthase (FASN)—a key enzyme for de novo synthesis of fatty acids [32]. Mechanistically, FASN SUMOylation is suggested to block its degradation by the proteasome system. FASN is highly expressed in many types of lung cancers [112]; thus, its stabilization by SUMO underscores a possible mechanism of upregulating fatty-acid synthesis by SUMOylation as part of the oncogenic process. Another case in which SUMOylation contributes to the stability of a protein related to lipid metabolism during cancer development is the SUMOylation of the transcription factor FOXA2 in the rat insulinoma-cultured cell line, INS-1E [113,114]. The Ubc9 and PIAS1-dependent conjugation of SUMO1 on lysine 6 leads to FOXA2 stabilization and the upregulation of FOXA2 target genes. Similarly, SUMO controls the localization and stability of another member of the forkhead family of transcription factors, FOXL2, a regulator of sterol metabolism [115,116]. Whether the SUMOylation of FOXA2 or FOXL2 directly affects lipid metabolism as part of the mechanism underlying cancer development is yet to be determined.

## 12. Cholestasis

Cholestasis is a disease of impaired bile-acid metabolism of the liver and bile-excretory system. One recent study in a mouse model system has demonstrated a functional link between the activity of SUMO and cholestasis [30]. An excess of bile acids induces the colocalization of the SUMO E3 ligase, RANBP2, with the Small Heterodimer Partner protein (SHP) on the nuclear membrane of cells [30]. The conjugation of SUMO by RANBP2 to lysine 68 of SHP facilitates its transport to the nucleus. Nuclear SHP forms a complex with histone modifiers that inhibit transcription in order to downregulate bile acids synthesis. Supporting this finding, mice expressing a SUMOylation-deficient SHP variant have increased levels of bile acid in the liver, and upon biliary insults, these mice developed cholestatic pathologies [30]. This report places SUMO as a critical regulator of the negative feedback loop controlling the balance between bile-acid synthesis and clearance and provides a possible SUMO-related mechanism for cholestasis.

## 13. Familial Partial Lipodystrophy

Familial Partial LipoDystrophy (FPLD) is a metabolic disorder of adipocyte degeneration after puberty. A subset of patients, with the Dunnigan-type FPLD, have mutations in the LMNA gene, which encodes the intermediate filament proteins lamins A and C [117]. Notably, at least three of the FPLD-causing mutations reduce the level of lamin A SUMOylation [118]. Although the link between the SUMOylation of lamins and FPLD is not entirely clear, several FPLD-causing mutations also decrease the level of binding of lamin A to the transcription factor SREBP1. The impaired binding of lamin A and SREBP1 alters the expression of a subset of SREBP1 target genes [119]. Based on these findings, a model was proposed in which changes in the level of lamin A SUMOylation impair critical aspects of SREBP1 activity [118]. This impairment leads to the misregulation of lipid-related programs and subsequently the development of familial partial lipodystrophy. Whether impaired SUMO activity is a cause or result of the development of the disease is an exciting topic for future investigation.

## 14. Concluding Remarks and Perspectives

In recent years, the possible role of SUMO as a regulator of lipid metabolism has been established, with specific implications for health and disease. So far, much of what we know about the role of SUMO in lipid metabolism comes from studies of mammals. Nevertheless, the basic components and regulatory circuits of lipid metabolism are conserved from yeasts to humans. Therefore, the study of nonmammalian model organisms that have well-defined physiology and tractable genetics are expected to deepen our understanding of the link between SUMO and lipid metabolism [9]. In mammals, our knowledge of how SUMOylation controls lipid metabolism is limited to the regulation of the metabolism of fatty acids and cholesterol. It remains to be addressed whether this reflects a mechanistic preference of the SUMO system towards the regulation of fatty acid and cholesterol metabolism or represents a research bias toward these two types of lipids. Moreover, regulation of lipid metabolism by SUMO is manifested by the control of the activity of specific transcription factors, such as the SREBPs and nuclear hormones receptors (Figure 3 and Figure 4) [9]. In sharp contrast to transcriptional regulation, only a few cases were reported in which SUMO is conjugated and alter the activity of enzymes that play a direct role in lipid metabolism. Proteome-wide screens for SUMO targets, however, have identified many enzymes of lipid metabolism as candidate targets for SUMOylation. This identification highlights the possibility of direct regulation of specific reactions of lipid metabolism by SUMOylation. In many reported cases of SUMOylation, the dynamic and specificity of SUMO conjugation are determined by the activity of E3 ligases [9]. In agreement with this general notion, in several cases, specific E3 ligases, such as PIAS4 and PIAS1, mediate the SUMOylation of specific transcription factors that are regulators of lipid metabolism (e.g., Figure 3B). The identification of new targets for specific E3 ligases is expected to expand our understanding of the repertoire of SUMOylated proteins that are involved in lipid metabolism and to unveil the functional weight of transcriptional regulation versus direct SUMOylation of enzymes of lipid metabolism. Although the mechanistic details are often not entirely clear, impaired SUMOylation can result in the development of specific diseases of lipid metabolism, such as NAFLD. Understanding how dysregulated SUMOylation affects the etiology of such diseases is expected to open new avenues for the development of new therapeutic strategies for the treatment of these devastating diseases.
